# A Novel Approach Toward Fabrication of High Performance Thin Film Composite Polyamide Membranes

**DOI:** 10.1038/srep22069

**Published:** 2016-02-29

**Authors:** Behnam Khorshidi, Thomas Thundat, Brian A. Fleck, Mohtada Sadrzadeh

**Affiliations:** 1 Department of Mechanical Engineering, 10-367 Donadeo Innovation Center for Engineering, Advanced Water Research Lab (AWRL), University of Alberta, Edmonton, AB, Canada, T6G 1H9; 2 Department of Chemical & Materials Engineering, 13-287 Donadeo Innovation Centre for Engineering, University of Alberta, Edmonton, AB, Canada, T6G 1H9

## Abstract

A practical method is reported to enhance water permeability of thin film composite (TFC) polyamide (PA) membranes by decreasing the thickness of the selective PA layer. The composite membranes were prepared by interfacial polymerization (IP) reaction between meta-phenylene diamine (MPD)-aqueous and trimesoyl chloride (TMC)-organic solvents at the surface of polyethersulfone (PES) microporous support. Several PA TFC membranes were prepared at different temperatures of the organic solution ranging from −20 °C to 50 °C. The physico-chemical and morphological properties of the synthesized membranes were carefully characterized using serval analytical techniques. The results confirmed that the TFC membranes, synthesized at sub-zero temperatures of organic solution, had thinner and smoother PA layer with a greater degree of cross-linking and wettability compared to the PA films prepared at 50 °C. We demonstrated that reducing the temperature of organic solution effectively decreased the thickness of the PA active layer and thus enhanced water permeation through the membranes. The most water permeable membrane was prepared at −20 °C and exhibited nine times higher water flux compared to the membrane synthesized at room temperature. The method proposed in this report can be effectively applied for energy- and cost-efficient development of high performance nanofiltration and reverse osmosis membranes.

Water crisis, according to the Global Risks Report by World Economic Forum in 2015, is the foremost global risk to social, environmental, and economical development of many countries in the next ten years^1^. Over the past decade, demand for fresh water has drastically increased with rapid growth in the world’s population, advancement in industrialization, global climate change and growing scarcity of surface and ground water resources^2^. The lack of fresh water has thus accelerated efforts toward improvement of the current treatment processes and development of novel techniques to sustainably produce potable water from sea water desalination and industrial and municipal waste waters reclamation^3^.

Membrane separation technologies, mainly nanofiltration (NF) and reverse osmosis (RO), have secured an important role in available water purification processes as a promising single step technique for removing multiple sized solutes and organic pollutants from contaminated water. Currently, most of commercial desalination plants employ RO and NF with thin film composite (TFC) membranes at the heart of the separation processes^4^,^5^. The TFC membranes are also widely-used in other membrane-based filtration applications including food, pharmaceutical and chemical industries^6^,^7^. These membranes typically consist of at least two compositional layers, (i) a top thin selective layer and (ii) a bottom porous sublayer which are of different structures and materials^8^. The porous support provides the required mechanical stability for the whole membrane structure to operate under high pressures while the ultrathin top layer plays the principal role in water filtration. The selective thin layer is typically fabricated from polyamide (PA) using an *in-situ* interfacial polymerization (IP) reaction between two reacting monomers (diamine and polyacyl chloride) at the surface of a porous (polysulfone or polyethersulfone) support. The multilayer feature of TFC membranes exploits the highly desirable advantage that each layer in the composite membrane can be independently optimized with the proper choice of materials and preparation methods for the specific application of interest^9^.

In general, conventional membranes are subject to a trade-off relationship between permeability and selectivity, i.e. high flux membranes show a low rejection percentage and vice versa. Therefore, one of the hoped-for goals of the research in the field of membrane fabrication has always been to develop “super-flux” membranes with high separation efficiency. Recent advances were focused on improving the membrane synthesis protocols^10^,^11^, modifying the surface properties by grafting additional polymer onto the surface^12^,^13^ and on developing nanocomposite membranes by the incorporation of hydrophilic metal oxide nanofillers, nanoporous zeolite particles, carbon nanotubes and graphene oxide nanosheets^14^,^15^,^16^,^17^,^18^,^19^. Although most of these efforts have shown promising results in the lab-scale, they are still faced with the challenges of cost-efficient synthesis process and easy scale-up for high volume industrial practices^20^.

Here we report a novel, simple and efficient method to enhance the water permeation of the TFC membranes by synthesizing the PA film at sub-zero temperatures of organic solution. Although extensive research has been carried out on the effects of influential synthesis parameters such as concentration of monomers, reaction time, curing temperature and time^21^,^22^,^23^,^24^,^25^,^26^, investigation of the effect of organic solution temperature has been limited to a few studies. Ghosh *et al*.^25^ investigated the effect of variation in the temperature of the organic solution from 8 °C to 38 °C, on the surface morphology and permeation performance of the TFC membranes. The TFC PA membranes were made by IP reaction between m-Phenylenediamine (MPD) in water and trimesoylchloride (TMC) in Isopar-G solution. It was demonstrated that the synthesis of the TFC membranes at lower temperatures decreased water permeation of the membranes by formation of thicker and denser PA films. In contrast, at higher temperatures of organic solution, thinner, rougher and more water permeable films with higher hydrophilicity were produced. Yu *et al*.^26^ reported the same trend for their synthesized TFC membranes by the reaction of MPD-aqueous solution and 5-chloroformyloxyisophthaloyl chloride (CFIC)-Isopar-G solution at different temperatures ranging from 10 °C to 40 °C. However, the aforementioned studies have not captured the influence of the organic solution temperature, which becomes critical at sub-zero temperatures.

In the present work, the variation range of organic solution temperature is broadened from −20 °C to 50 °C using three different solvents (hexane, cyclohexane and heptane). It was anticipated that the change in the temperature of the organic solution would change the transfer rate of the monomers toward the reaction zone and thus would alter the rate of polymerization reaction, final physico-chemical and permeation properties of the resulting TFC membranes. The prepared TFC membranes were characterized using scanning electron microscopy (SEM), transmission electron microscopy (TEM), X-ray photoelectron spectroscopy (XPS), Fourier transform infrared spectroscopy (FTIR), atomic force microscopy (AFM) and contact angle measurement. Finally, the improved performance of the TFC membranes (water flux and salt rejection) was correlated to changes in the chemical and morphological structures of the selective layer which were imparted by the variation of the organic solution temperature.

## Results

**Figure 1:**
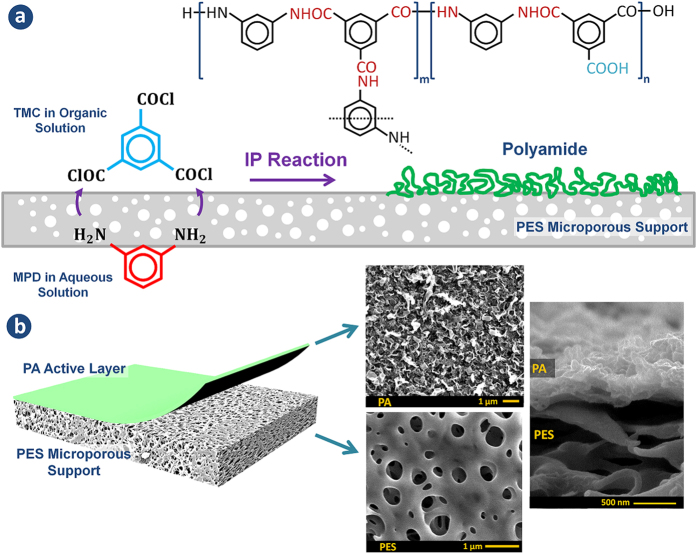
(**a**) Schematic representation of the interfacial polymerization reaction between MPD and TMC at the surface of the microporous PES support and the chemical formula of PA layer. The m and n in polymer structure represents the crosslinked and the linear parts, respectively (m + n = 1). (**b**) Structure of the synthesized TFC membranes with the top and cross-sectional morphologies.

The PA skin layer in this study was synthesized by *in-situ* IP reaction between MPD monomer in water and TMC monomer in organic solvent. By bringing the two immiscible water and organic solutions into contact at the surface of the PES support, the reacting MPD and TMC monomers start partitioning to the water-organic interface and form the PA selective layer. The schematic view of the IP reaction and the chemical formula of the PA polymer are presented in Fig. 1a. The resulting TFC membranes consist of two separate layers: an ultrathin PA selective layer (~50–400 nm) over a microporous PES substrate (~140 μm), as shown in Fig. 1b.

Before proceeding to examine the effect of the organic solution temperature on the properties of the PA TFC membranes, it is necessary to understand the mechanism of the IP reaction. The reaction between a diamine and acid chloride is a common condensation polymerization reaction that can be homogenously completed in a suitable reactor to yield PA and HCl as a by-product. However, the formation of PA by *in-situ* IP reaction adds more complexity to the kinetics of polymerization reaction, i.e. the rate of the monomers mass transfer which directly changes the concentration of the reactive monomers in the reaction zone and thus affects the polymerization rate and final properties of the TFC membranes^21^,^27^,^28^. It is believed that formation of the PA film by the IP reaction does not follow a uniform growth. The early stage of the IP reaction is markedly fast, resulting in an ultrathin PA (incipient) film at the PES surface. Subsequently, the IP reaction shifts to a slower growth stage as the incipient PA film hinders the diffusion of the remaining MPD molecules to the reaction zone. The polymerization eventually stops when the mass transfer resistance of the existing PA layer becomes larger than the driving force for the MPD diffusion to the reaction zone. Regarding that, the PA growth at the support surface is considered as a self-limiting diffusion-controlled process^29^,^30^. It is also worth noting that the IP reaction mainly happens at the organic side of the interface due to higher solubility of the MPD molecules in the organic phase compare to that of TMC in water^31^. Therefore, any variation in the thermodynamic properties of the organic solution (especially surface tension and viscosity) is expected to change the solubility, diffusion and partitioning of the MPD molecules into the reaction zone which consequently changes the rate of the polymerization reaction, the final surface morphology and permselectivity of the TFC membranes.

The surface tension and viscosity of three organic solvents namely cyclohexane, hexane and heptane at different temperatures are presented in Table S1 in the “Supplementary Information” section^32^,^33^,^34^,^35^. For all solvents, both the surface tension and viscosity decrease with increase in temperature and vice versa. The observed change in these properties is believed to be the principle reason for the substantial change in the final chemical and physical characteristics of the polymerized film. Turning now to the experimental evidence on this change, morphological and physico-chemical characterization of the synthesized membranes are presented.

**Figure 2:**
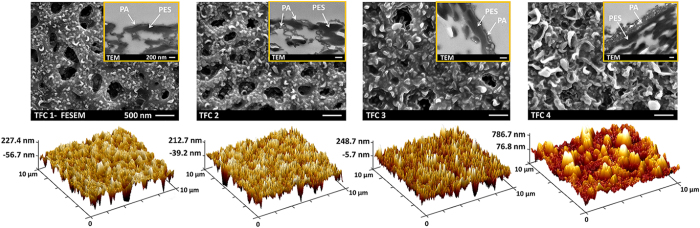
Surface FESEM, cross-sectional TEM and 3D AFM images of the TFC membranes. The synthesis conditions were the same for all TFC membranes except the temperature of the heptane solution which was −20 °C for TFC1, 1 °C for TFC 2, 25 °C for TFC 3 and 50 °C for TFC 4. Detailed information about the synthesis process is presented in the “Materials and Methods” section.

Figure 2 illustrates the FESEM, TEM and AFM images of four TFC membranes which were prepared in heptane at different temperatures (The FESEM images of the TFC membranes prepared in cyclohexane and hexane are presented in Supplementary Information). These images provide useful information about the surface structure, thickness and roughness of the PA films, respectively. As can be seen, the surface morphology of the PA skin layer is noticeably different for the 4 membranes. The surface of the TFC 3 membrane, which was prepared at 25 °C, has several wrinkled shapes which is well-known as a “ridge-and-valley” structure^36^. By increasing the organic solvent temperature (see TFC 4 which is prepared at 50 °C), the wrinkled protuberances enlarged and resulted in a thicker PA layer as it is more evident in the TEM cross-section image. In contrast, for the membranes synthesized at lower temperatures (see TFC 1 & 2 which are prepared at −20 °C and 1 °C, respectively), the size of the ridges and valleys decreased remarkably and a thinner PA film was produced at the surface. The 3D AFM image of TFC 4 membrane also confirmed the formation of rougher PA films at high temperature of organic solution compared to the other membranes. It is worth mentioning that the apparent holes in the FESEM image of TFC 1 and TFC 2 correspond to PES substrate, not the PA skin layer (see the FESEM image of the base PES support in the Fig. 1b). These holes are visible in TFC 1 and TFC 2 due to formation of an ultrathin layer of the PA on the support surface, whereas they are completely covered by a thicker PA film in TFC 3 and TFC 4. Taking a closer look at the TEM images of TFC 1 and TFC 2 in Fig. 2, it is found that the support holes are internally closed by the PA layer otherwise the rejection percentage would fall down drastically.

**Figure 3:**
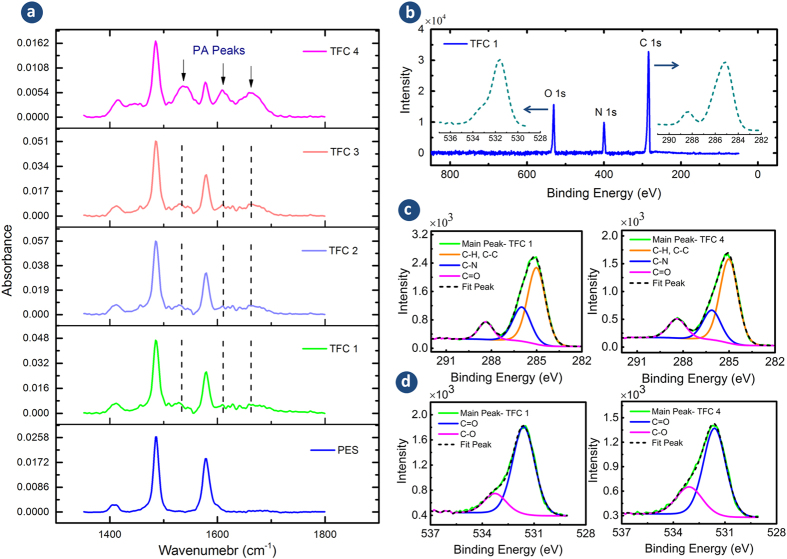
(**a**) FTIR spectra (PES support and TFC1-4), (**b**) XPS survey spectrum (TFC 1) along with high resolution C (1s) and O (1s) spectra, (**c**) convoluted high resolution C (1s) and (**d**) convoluted high resolution O (1s) spectra (TFC 1 & TFC4). FTIR shows additional peaks associated with the PA to the PES support. The survey spectrum indicates the presence of O, N and C elements and the absence of S on the surface of the membranes indicating all membranes are integrally skinned. The convoluted high resolution C (1s) and O (1s) peaks provide information about the PA chemical bonds that helps to quantify C=O/C−N ratio. The high resolution C (1s) and O (1 s) spectra of TFC 2 and TFC 3 membranes are presented in Supplementary Information.

In order to provide the information about compositional elements and functional groups of the polyamide surface, FTIR and XPS analyses were conducted. The FTIR spectra of the TFC 1–4 membranes (Fig. 3a) confirm the successful formation of a PA skin layer at the surface of the PES support by the *in-situ* IP reaction. According to this figure, the FTIR spectrum of the base PES substrate had three peaks at 1410, 1485, and 1580 cm^−1^ due to the aromatic ring (benzene) vibration^37^,^38^. However, for the composite membranes, three new peaks at 1541, 1611, and 1667 cm^−1^ were identified which are attributed to the PA skin layer over the PES support. These peaks are related to C=O stretching of the amide I bond, aromatic amide ring breathing and N–H bending of amide II in the –CO–NH– group, respectively^39^,^40^,^41^,^42^.

XPS analysis was carried out to evaluate the elemental composition, chemical bonding and degree of cross-linking of the top 5–10 nm of the PA active layer. The absence of a sulfur peak, which is the principle peak of the PES support, implies the formation of an integrally skinned PA layer at the support surface for all TFC membranes. The XPS survey spectra of TFC 1, which has the thinnest PA skin layer (Fig. 3b), shows the presence of only three elements, namely oxygen (O 1s), nitrogen (N 1s) and carbon (C 1s) at the membrane surface. The XPS survey spectra of the other TFC membranes were similar to TFC 1 and thus were not shown here.

XPS analysis provides atomic concentration of elements at the surface which can be used to evaluate the degree of cross-linking of the PA layer by the following equation^43^.




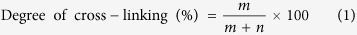




where *m* and *n* are the cross-linked and the linear part of the PA layer as shown in Fig. 1. The values of m and n can be calculated based on experimental O/N ratio obtained from XPS analysis by^21^,^43^.




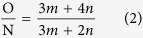




Theoretically, O/N ratio varies between 1.0 for fully cross-linked (C_18_H_12_N_3_O_3_, *n* = 1 and *m* = 0) and 2.0 for fully linear (C_15_H_10_O_4_N_2_, *n* = 0 and *m* = 1) structure. Table 1 summarizes the XPS analysis of the synthesized TFC 1–4 membranes. The results of the elemental compositions show that by decreasing the temperature of the organic solution, the experimental O/N ratio nears the theoretical values for fully cross-linked PA films (~1.0) which is comparable with that of commercial XLE, LE, ESPA3 and SWC4 Hydranuatics membranes^40^. In contrast, by elevating the organic solution temperature to 50 °C, the O/N ratio of the synthesized TFC membranes increased to 1.42, implying formation of more linear structures in the PA network similar to commercial NF90 and FT-30 Filmtec membranes^44^,^45^.

**Figure 4:**
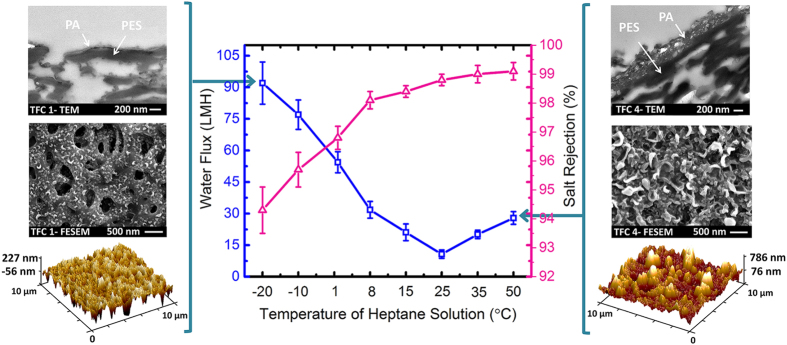
Water flux and salt rejection of the TFC membranes prepared at different temperature in 0.2 wt.% TMC-heptane solution. The surface and cross-sectional images of the membranes synthesized at −20 °C and 50 °C are presented to justify the permeation properties. Test conditions: feed solutions: pure water and 2000 ppm NaCl solution, pressure: 1.52 Mpa (220 psi), temperature: 25 °C, pH: 6.5–7.

The information of chemical bonding was obtained by deconvolution of C (1s) and O (1s) high resolution XPS spectra as shown in Fig. 3c. The C (1s) high resolution spectra for all TFC membranes showed three peaks: a major peak at 285 eV which is assignable to a carbon atom without adjacent electron withdrawing atoms (carbons in aliphatic/aromatic C−C and C−H), an intermediate peak at 286.5 eV that is associated with carbon in weak electron withdrawing atoms (carbons in C−N) and a minor peak at 288.5 eV which corresponds to carbons attached to strong electron withdrawing atoms (carbons in carboxylic O=C−O and amides O=C−N)^46^. The C=O/C−N ratio can also be used for comparing the degree of cross-linking of the synthesized membranes. Theoretically, for every aromatic carbon attached with a single bond to nitrogen there is one carbon in amide group which makes the O=C−N/C−N ratio equal to 1.0. Therefore, for a highly cross-linked PA film, the C=O/C−N and O=C−O/O=C−N must approach 1.0 and 0.0, respectively, due to the lower number of carboxylic groups in the polymer structure. According to Table 1, it can be concluded that the membranes prepared at lower temperatures of the organic solution had a higher degree of cross-linking. High resolution O (1s) spectra have two peaks (Fig. 3c), which illustrate the presence of two types of oxygen in the PA layer: O=C at 531.6 eV and O−C at 533.0 eV^47^,^48^. The ratio of O=C/O−C for TFC 1–4 membranes was calculated to be 4.53, 3.35, 3.04, and 2.78, respectively. This again confirms that synthesis of TFC membrane at low temperatures results in more cross-linking in the PA network, while elevating the reaction temperature decreases the cross-linking density of the PA layer. Table 2 presents the contact angle and surface roughness of TFC1-4 membranes. Contact angle measurement is commonly carried out for evaluating the wettability of the membrane surface. A lower value of the contact angle often enhances water permeation through the membrane^49^. The contact angle measurement is influenced by both surface chemical composition (hydrophilic/hydrophobic functional groups) and morphology (roughness)^50^. Since the chemical composition of the TFC1-4 membranes were similar, as suggested by the FTIR spectra in Fig. 3a, the change in the contact angle values is mainly attributed to the different surface morphologies of these membranes. Compared to TFC 3 which had the contact angle of 81.2°, the other membranes had a substantially lower contact angle, suggesting that the variation of temperature of the organic solution (either decrease or increase from the 25 °C) resulted in a TFC membrane with higher wettability. The average roughness (*R*_a_) and the root mean square (*R*_q_) roughness data in Table 2 show that the TFC 1 to 3 membranes, prepared at −20 °C, 1 °C and 25 °C, respectively, had comparable surface roughness. However, the roughness of TFC 4 membrane (prepared at 50 °C) was notably higher (almost three times) than the other membranes, implying that synthesis of TFC membrane at higher temperature of organic solution will increase the surface roughness of the resulting PA film. Figure 4 presents the water flux and NaCl rejection of eight TFC membranes which were synthesized in heptane solutions at different temperatures. The water flux and salt rejection of the TFC membranes prepared using cyclohexane and hexane are provided in Supplementary Information. According to Fig. 4, the TFC membrane prepared at 25 °C (TFC 3) had the lowest water flux among the other synthesized membranes with 10.7 LMH and 98.8% salt rejection. The water permeation improved significantly to 92.1 LMH with just 4% sacrifice in rejection percentage when the TFC membrane was formed using TMC-heptane solution at −20 °C. Moderate enhancement in water flux and salt rejection up to 27.9 LMH and 99.1%, respectively, was also observed by increasing the organic solvent temperature from 25 °C to 50 °C. The permeation properties of the two most permeable TFC membranes, prepared at −10 °C and −20 °C, are compared with three commercially available RO membranes namely Filmtec BW30, TriSep X-20, and Hydranautics ESPA membranes in Table 3. The experimental results show that the sub-zero lab-made TFC membranes provided higher water flux than commercial RO membranes with comparable salt rejection percentage.

## Discussion

**Figure 5:**
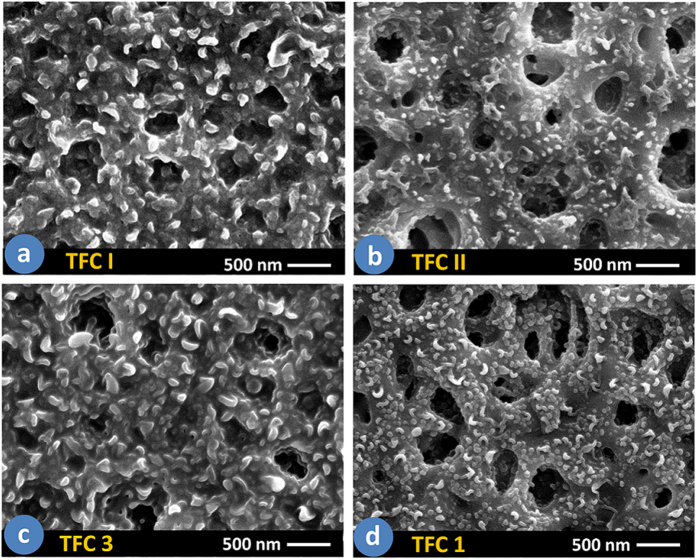
FESEM images of (**a**) TFC I membrane prepared with 2 wt. % MPD and 0.15 wt. % TMC, NH_2_/COCl = 21.1, in hexane at 25 °C; (**b**) TFC II membrane prepared with 2 wt. % MPD and 0.35 wt. % TMC, NH_2_/COCl = 9.0, in hexane at 25 °C; (**c**) TFC 3 membrane prepared with 2 wt. % MPD and 0.2 wt. % TMC, NH_2_/COCl = 15.8, in heptane at 25 °C; and (**d**) TFC 1 membrane prepared with 2 wt. % MPD and 0.2 wt. % TMC, NH_2_/COCl = 15.8, in heptane at −20 °C.

The significant change in the surface characteristics of the TFC membranes due to the variation of organic solution temperature can be attributed to the changes in the solubility and diffusivity of the MPD molecules into the reaction zone. At higher temperatures of the TMC-organic solution, the surface tension and the viscosity of the organic solvent decrease (see Table S1) which allows the MPD molecules to have more solubility and diffusivity into the organic phase. Additionally, more swelling of the initially formed PA layer at high temperature will facilitate migration of the MPD molecules from the aqueous solution to organic phase. The more available MPD molecules in the reaction zone increases the amine to acyl chloride molar ratio (NH_2_/COCl) and thus speeds up the rate of polymerization and produce thicker PA film at the surface. Furthermore, higher thermal energy, imparted by surrounding organic solvent to the MPD molecules, increases their local movement to reach the TMC-rich spots in the reaction zone which results in formation of larger ridges and valleys and thus rougher PA film as it is observable in the case of TFC 4 which was prepared at 50 °C. However, the miscibility of water and organic solvent increases with increase in temperature^51^,^52^,^53^,^54^. Since the TMC molecules can be readily hydrolyzed by water, the diffusion of water molecules into the reaction zone is considered as an important competitive reaction which alters the cross-linking density of the PA film by reducing the number of reacting carboxyl groups of TMC^29^. Therefore, the resulting PA layer has less extent of cross-linking as confirmed by C=O/C–N and O/N ratio in Table 1 for TFC 4. In contrast, at very low temperatures of organic solution, the MPD solubility and diffusivity into the reaction zone decreases due to the higher surface tension and viscosity of the solvent. Furthermore, the low temperature of organic solution quenches the incipient PA layer and hinders further diffusion of the MPD molecules from aqueous solution to organic phase. Therefore, the transport rate of the MPD molecules to the organic side of the interface and thus the ratio of available amine/acyl chloride in the reaction zone decreases. As a result, a thinner PA skin layer with smaller ridges and valleys, more cross-linking density and higher water permeation forms at the interface (see the properties of TFC1 and TFC 2 in Figs 2 and 4). It is worth noting that the changes in amine/acyl chloride ratio in the reaction zone due to different organic solution temperature is very similar to the changes imparted by the different initial monomer concentration in water and organic solutions, reported earlier by the authors^21^. Figure 5a,b illustrate the surface morphology of the TFC I and TFC II membranes which were prepared in hexane at room temperature (25 °C) but with different MPD and TMC concentrations (thus different amine/acyl chloride ratio). For comparison, the surface images of the TFC 3 and TFC 1 membranes which were prepared at different organic solution temperature but with identical monomer concentration are presented in Fig. 5c,d. According to Fig. 5a, at higher amine/acyl chloride ratio (TFC I, NH_2_/COCl = 21.1), the PA surface has the wrinkled ridges and valleys, similar to the surface structure of TFC 3 (Fig. 5d, membrane prepared at 25 °C with NH_2_/COCl = 15.8). However, when the amine/acyl chloride ratio decreased in TFC II (Fig. 5b, membrane prepared at 25 °C with NH_2_/COCl = 9.0), the structure noticeably changed to a fine morphology with small micro-protuberances which is quite similar to the surface morphology of TFC 1 synthesized at −20 °C (Fig. 5d, NH_2_/COCl = 15.8).

The above described changes in physico-chemical properties of the PA active layer manifest their effects through altering the permeation performance of the synthesized TFC membranes. In general, water permeability of a TFC membrane is strongly related to both structural (thickness, density, and pore size) and surface (roughness and hydrophilicity/hydrophobicity) properties of the membrane^55^. Since, the water flux of all synthesized membranes followed a curve with a minimum at room temperature (see Fig. 4), it can be concluded that there exist competing factors and the final water permeation of the membrane is affected by a trade-off relationship between these factors. Although the TFC membranes prepared at sub-zero temperatures of organic solutions showed higher degree of cross-linking, the water flux enhanced significantly due to a remarkable decline in the thickness of the PA active layer. The consistent decrease in water flux with increases in organic solution temperature up to 25 °C due to formation of thicker PA layer is an evidence for dominant impact of the PA thickness on water flux. The moderate increase in water flux for the TFC membranes prepared above room temperatures, might be attributed to the presence of larger ridges and valley at the surface which may contribute to the enhancement of water permeation by providing more effective contact area between water molecules and membrane surface^56^.

These observations clearly demonstrate the significant effect of the organic solution temperature on the physico-chemical characteristics of the synthesized TFC membranes. The results can be easily employed (i) to fabricate cost-efficient TFC membrane by eliminating the requirement for high concentration of monomers, (ii) to facilitate robust fabrication of high-flux membranes by replacing two influential factors (concentrations of both monomers) with one factor (temperature of organic solution) in the membrane synthesis process^21^. The latter reduces the uncertainties associated with fabrication of the TFC membranes and consequently increases the repeatability (which commercially equates to quality) of membrane properties.

## Materials and Methods

### Materials and chemicals

MPD (≥99%) and TMC (98%) were obtained from Sigma-Aldrich and used as reacting monomers. Hexane (≥99%) and heptane (≥99%) were purchased from Fisher Scientific and used as organic solvents. Sodium dodecyl sulfate (SDS), triethylamine (TEA) and camphorsulfonic acid (CSA) were obtained from Sigma-Aldrich and utilized as additives in the aqueous solution. All the materials were used as they were received without further purification. Microporous polyethersulfone (PES, 0.2 μm) was purchased from Sterlitech and used as support.

### Synthesis of PA TFC membranes

The PA TFC membranes were prepared via interfacial polymerization (IP) reaction between MPD and TMC at the surface of the PES support. First, the PES microporous sheet was dip-coated in the MPD-aqueous solution (2 wt.% MPD, 0.2 wt.% SDS, 2 wt.% CSA and 1 wt.% TEA) for 15 minutes. The PES substrate was then removed and the excess amine solution was removed from the surface using a rubber roller. Afterwards, the impregnated PES support was brought into contact with TMC-organic (cyclohexane, hexane, and heptane) solution for 30 seconds to allow the polymerization reaction at the surface. The temperature of the organic solution was changed using isotemperature water bath (Isotemp 3013, Fisher Scientific) and freezer (Fisher Scientific, Isotemp™ freezer). The organic solution was kept in sealed glass vials to ensure no loss of solvent and change in monomer concentration at elevated temperatures. During the course of polymerization, the PES substrate was fixed within a plexiglass acrylic frame so that only the top surface of the PES support was in contact with organic solution. The resulting TFC membranes were then thermally treated in a digital oven (Thermo Scientific Heratherm™, USA) at 70 °C for 5 min. Finally, to remove any residual solution from the surface, the TFC membranes were washed 10 times with 250 mL deionized (DI) water and kept in the DI water bath at room temperature for characterization tests.

### TFC membranes characterization

The surface morphology of the TFC membranes was analyzed using field emission scanning electron microscopy (FESEM, JEOL 6301 F). The membranes were sputter coated with a thin layer of chromium and imaged at an accelerating voltage of 5.0 kV and magnification of 30,000×.

The ultrathin cross-sectional images of the TFC membranes were obtained using transmission electron microscopy (TEM, Philips/FEI Morgagni 268, The Netherlands) at acceleration voltage of 80 kV. The sample preparation protocol included first staining in uranyl acetate and lead citrate, then embedding in spurr’s resin, and finally sectioning using ultramicrotome (Reichert-Jung Ultracut E, USA).

Attenuated total reflection Fourier-transform infrared (ATR-FTIR, Thermo Nicolet Nexus 670, USA) spectroscopy was used to determine the functional groups associated with the top few microns of the synthesized membranes. The FTIR spectra of the TFC membranes were averaged from 512 scans and were taken over the range of 600–4000 cm^−1^ at 4 cm^−1^ resolution.

The elemental composition (C, O, N) of the top 5–10 nm of the PA skin layer was identified using an X-ray photoelectron spectroscope (XPS, Kratos AXIS ULTRA, UK) equipped with a monochromatic Al Kα X-ray source. Survey spectra were collected at constant pass energy of 160 eV, with a scan step size of 0.4 eV, and sweep time of 100 s in the range of 0–1100 eV. High resolution spectra for C, O and N elements were collected with pass energy of 20 eV, step size of 0.1 eV, and sweep time of 200 s. The measured binding energies were calibrated with respect to C1s hydrocarbon bond at 284.6 eV.

Atomic force microscopy (AFM, Bruker Dimension Icon, USA) was used to capture the surface topography of the TFC membranes. An area of 5 μm × 5 μm of the TFC membranes was scanned three times using tapping mode at scan rate of 1.0 Hz at ambient conditions of temperature and humidity. Nanoscope analysis software V.1.40 was used for processing the AFM data, removing the noise and calculating the average (*R*_a_) and the root mean square (*R*_q_) roughness values.

The surface wettability of TFC membranes was evaluated through contact angle measurements using Krüss DSA 100 instrument (Krüss GmbH, Germany). A sessile drop of ultra-pure water was placed on the surface of the TFC membranes and the static contact angle was measured. The contact angle was measured at 5 different locations for each sample in order to minimize the experimental error.

The permeation properties of the TFC membranes were evaluated using a cross flow filtration unit (Sterlitech Co., CF042A cell, USA) at a trans-membrane pressure of 1.52 MPa and at a feed flow rate of 1 L min^−1^. Detailed explanation of the membrane filtration unit is presented in Supplementary Information section. The water flux (*J*_W_) of the TFC membranes was obtained at steady state by measuring the volume of water (Δ*V*) passed through the effective filtration area (*A*) of the membrane during the elapsed time period of sample collection (Δ*t*):




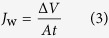




The apparent salt rejection (R) was calculated by




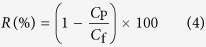




where *C*_p_ and *C*_f_ are the NaCl concentration in permeate and feed (2000 ppm NaCl) solutions, respectively, measured after 3 h filtration process^57^. Pure water permeability coefficient (*A*_P_) was determined at different applied pressure (1.52, 1.24, 0.96, and 0.69 MPa) and salt permeability coefficient (*B*) was calculated by 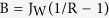 at 1.52 Mpa and 2000 ppm NaCl solution.

## Additional Information

**How to cite this article**: Khorshidi, B. *et al*. A Novel Approach Toward Fabrication of High Performance Thin Film Composite Polyamide Membranes. *Sci. Rep.* **6**, 22069; doi: 10.1038/srep22069 (2016).

## Supplementary Material

Supplementary Information

## Figures and Tables

**Table 1:**
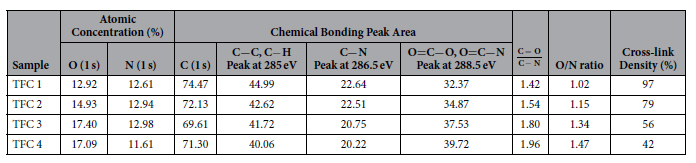
Elemental compositions, O/N ratio, chemical bonding peak area and degree of cross-linking of the TFC 1–4.

**Table 2:** Contact angle and surface roughness of synthesized TFC membranes.

Sample	Contact Angle (°)	Surface Roughness (nm)	
		R_a_	R_q_
TFC 1	53.3 ± 1.2	49.5 ± 2.1	65.1 ± 2.2
TFC 2	56.9 ± 1.1	54.2 ± 2.3	71.0 ± 3.8
TFC 3	81.2 ± 1.6	53.0 ± 1.6	66.3 ± 2.4
TFC 4	66.2 ± 1.0	130.7 ± 15.2	168.8 ± 12.1

**Table 3:** Permeation properties of the commercial RO membranes compared with the lab-made TFC membranes.

Membrane	Water Flux (LMH)	Salt Rejection (%)	*A*_P_ (LMH/bar)	B (LMH)
Filmtec BW30	69.7	94.3	4.26	3.54
Hydranautics ESPA	59	95.1	3.7	2.4
TriSep X-20	41.1	93.2	2.4	2.1
Lab-made TFC prepared at −10 °C	77	95.7	4.8	2.8
Lab-made TFC prepared at −20 °C	92	94.3	5.78	4.96

Test conditions: feed solutions: pure water and 2000 ppm NaCl solution, pressure: 1.52 Mpa (220 psi), temperature: 25 °C, pH: 6.5–7.
