# Supplementary material for: A Novel Approach Toward Fabrication of High Performance Thin Film Composite Polyamide Membranes
Source: Sci Rep. 2016 Feb 29;6:22069. doi: 10.1038/srep22069 (PMC4770410; doi:10.1038/srep22069)
Supplement: Supplementary Information [file srep22069-s1.pdf]

Supplementary Information:

A Facile Approach towards Fabrication of High Performance Thin Film Composite Polyamide Membranes

Behnam Khorshidi[1], Thomas Thundat[2], Brian A. Fleck[1], Mohtada Sadrzadeh[1*]

[1]Department of Mechanical Engineering, 10-367 Donadeo Innovation Center for Engineering, Advanced Water Research Lab (AWRL), University of Alberta, Edmonton, AB, Canada, T6G 1H9, Tel: (780) 492-

[2]Department of Chemical & Materials Engineering, 13-287 Donadeo Innovation Centre for Engineering, University of Alberta, Edmonton, AB, Canada, T6G 1H9

[*] Corresponding Author: sadrzade@ualberta.ca

Summary: The supplementary materials presented here provide more information and details to the results and discussion in the body of the manuscripts. This section includes a table for selected thermodynamic properties of the organic solvents used for synthesis of the membranes, the FESEM of the TFC membranes prepared with hexane and cyclohexane solutions, high resolution XPS spectra for TFC 2 and TFC 3 membranes, the water permeability and salt rejection of the membranes prepared with hexane and cyclohexane solutions as well as the schematic view of the cross-flow filtration setup.

Properties of organic solvents: Table S1 presents the selected thermodynamic properties of the organic solvents, used for preparation of the TFC membranes. The values of surface tension and viscosity are presented at five different temperatures.

The data for density (at 25 °C), melting point, boiling point, surface tension (at 25 and 50 °C) and viscosity (at 25 and 50 °C) were obtained from "CRC handbook of Chemistry and Physics".[1]

The reported values for surface tension at 1, -10 and -20 °C were calculated by $\sigma = a - bT$ which is a least-squares correlation based on experimental data given by Jasper.[2] In this equation σ is the liquid surface tension, T is temperature (°C), a and b equal to 20.44 and 0.1022, respectively for hexane and 22.1 and 0.098, respectively for heptane. The obtained data are in good agreement with experimental data reported by Grigoryev et al.[3]

The values of viscosity at 1, -10 and -20 °C were obtained Data obtained based on the Orrick and Erbar equation:

$$\text{Ln}\left(\frac{\eta_l}{\rho_1 M} \right) = A + \frac{B}{T}$$

where η_l is the liquid viscosity (mPa.s), T is the liquid temperature (K), ρ_l is the liquid density at 20 °C (g/cm^3), M is the molecular weight (g/mol), $A = -(6.95+0.21N)$, $B = 275 + 99N$ and N is the number of carbon atoms in the molecule.[4]

It was explained in the manuscript that the variation of the surface tension and viscosity of the organic solution, especially at lower temperatures, remarkably changed the rate of polymerization, the surface morphology and thickness of the active layer and the final permeation properties of the resulting TFC membranes.

FESEM Images: The surface morphology of the TFC membranes prepared in hexane and cyclohexane is presented in Figures S1 and S2, respectively. Compared to the FESEM images of the TFC 1-4 (Figure 2 in the manuscript), similar trend of change for the surface morphology can be observed for TFC 5-8 and TFC 9-12 which were prepared with hexane and cyclohexane solutions, respectively. Similar to the FESEM images of the TFC 1-4 membranes (see Figure 2 in the manuscript), the polyamide ridges and valleys at the surface of the TFC 5-8 and TFC 9-12 membranes become larger and result in thicker and rougher layer over PES support. In contrast, when the temperature of organic solution decreased, the surface features become smaller and a thinner film was produced at the surface

High resolution XPS: The high resolution spectra of the C (1s) and O (1s) of TFC 2 and 3 membranes are presented in Figure S3. The C=O/C−N ratio, extracted from these graphs, was utilized for calculating the degree of cross-linking of the synthesized TFC membranes

Flux and Rejection: The water flux and salt rejection of the TFC membranes prepared with hexane and cyclohexane solution were shown in Figure S4. As can be observed, the effect of temperature on flux and rejection followed the same trend for the three solvents. Comparing the permeation properties of the all TFC membranes (see Figure 4 and Figure S4), it can be concluded that TFC membranes prepared in heptane solution exhibited slightly higher water flux and salt rejection.

Cross-flow Filtration Setup: Schematic view of the cross-flow membrane filtration setup is shown in Figure S5. The setup consists of a stainless steel feed tank, membrane cell (Sterlitech Corporation, USA), a constant flow diaphragm pump of maximum capacity 6.8 LPM (1.8 GPM) from Hydra-Cell, a chiller/heater (Isotemp 3013, Fisher Scientific) to maintain the feed temperature at 25 °C, a bypass valve and a back pressure regulator (Swagelok) to control applied pressure and cross flow velocities. A digital weighing balance (Mettler Toledo) was used to measure the permeate flow rate and the data were directly collected in a computer using LabVIEW (National Instruments) data acquisition software.

References

1. *CRC Hanbook of Chemistry and Physics*. (CRC Press).

2. Jasper, J. J. The surface tension of pure liquid compounds. *J. Phys. Chem. Ref. Data* **1,** 841 (1972).

3. Grigoryev, B. A., Nemzer, B. V., Kurumov, D. S. & Sengers, J. V. Surface tension of normal pentane, hexane, heptane, and octane. *Int. J. Thermophys.* **13,** 453–464 (1992).

4. Poling, B. E., Prausnitz, J. M. & O’Connell, J. P. *Properties of Gases and Liquids*. (McGraw Hill Professional, Access Engineering, 2001).

Table S1: Selected properties of the organic solvents used for making TFC membranes

Solvent	Chemical Formula	Density[1] (kg.m^{-3})	Melting Point[1] (°C)	Boiling Point[1] (°C)	Surface Tension (mN.m^{-1})					Viscosity (mPa.s)				
					-20[2]	-10[2]	1[2]	25[1]	50[1]	-20[4]	-10[4]	1[4]	25[1]	50[1]
Cyclohexane	C_6H_{12}	773.9	6.7	80.7	NA	NA	NA	24.16	21.26	NA	NA	NA	0.894	0.615
Hexane	C_6H_{14}	660.6	-95.3	68.7	22.48	21.46	20.34	17.89	15.33	0.48	0.42	0.38	0.3	0.24
Heptane	C_7H_{16}	679.5	-90.55	98.38	24.06	23.08	22	19.66	17.19	0.69	0.6	0.52	0.39	0.3

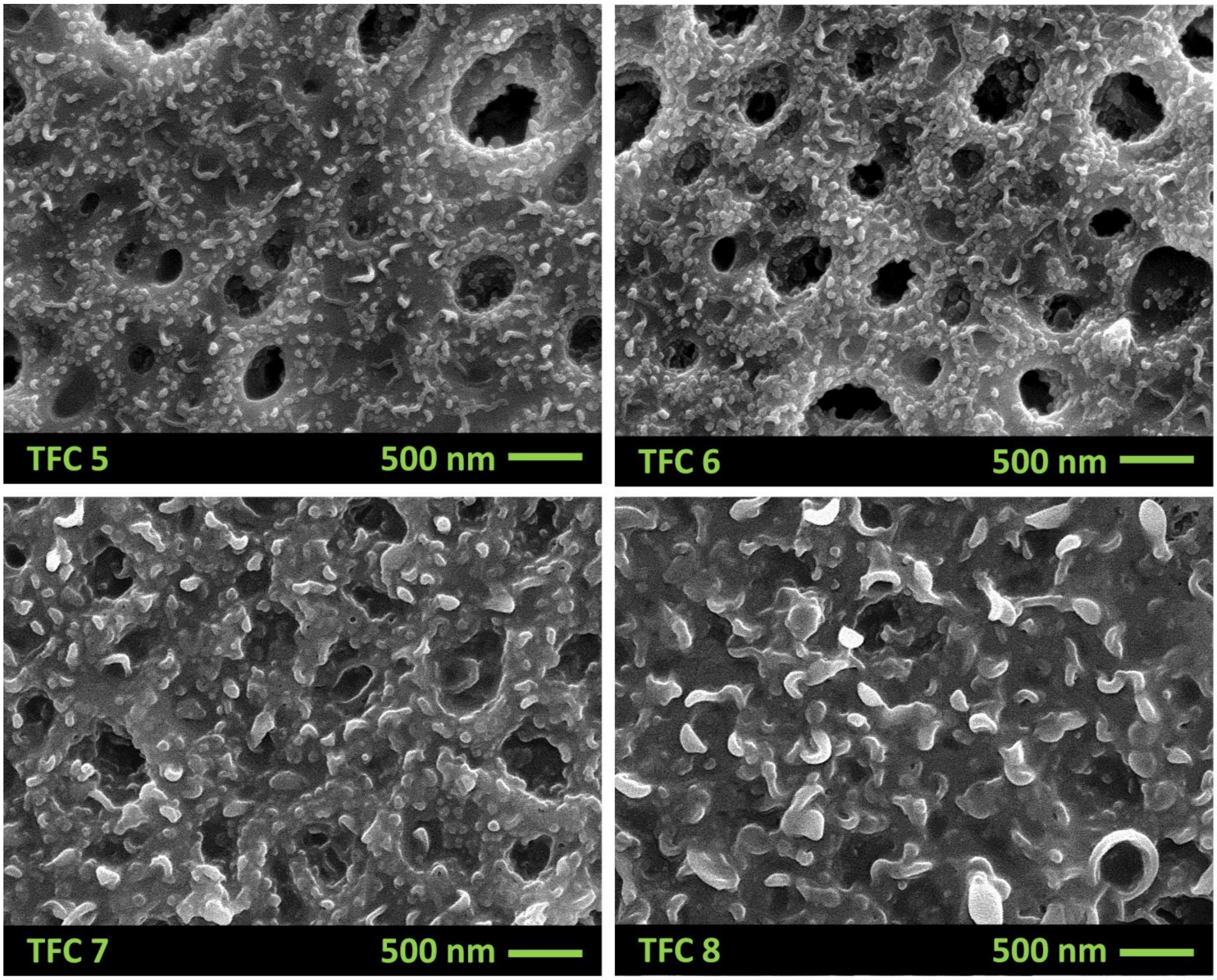

Figure S1: FESEM images of TFC membranes prepared in 0.2%-hexane solution. The temperature of the hexane solution for TFC 5 to 8 was changed as -20 °C, 1 °C, 25 °C and 50 °C respectively. All other synthesis conditions were the same as TFC 1-4 which were explained in “Materials and Methods” section

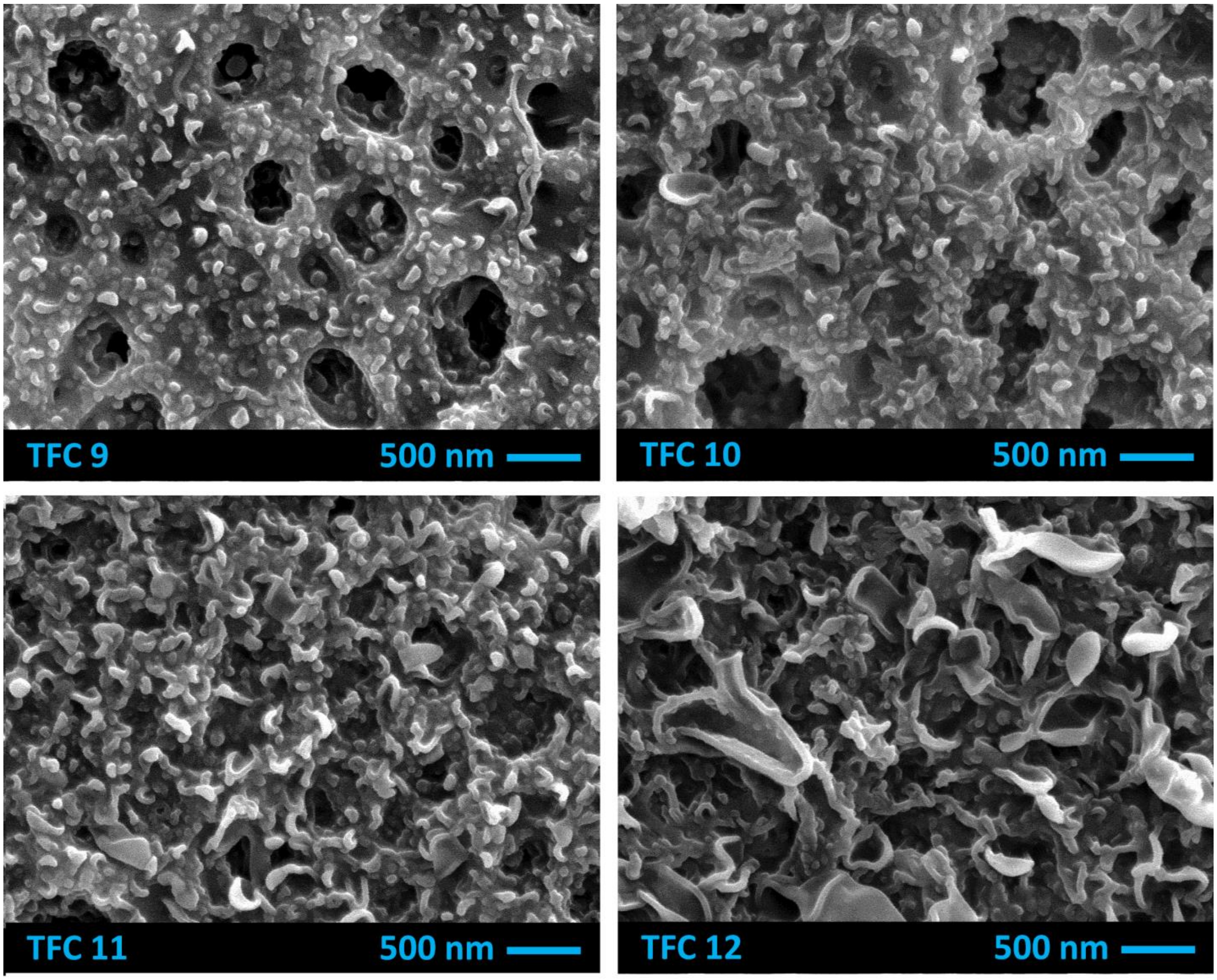

Figure S2: FESEM images of TFC membranes prepared in 0.2%-cyclohexane solution. The temperature of the cyclohexane solution for TFC 9 to 12 was changed as 8 °C, 25 °C, 35 °C and 50 °C respectively. All other synthesis conditions were the same as TFC 1-4 which were explained in "Materials and Methods" section

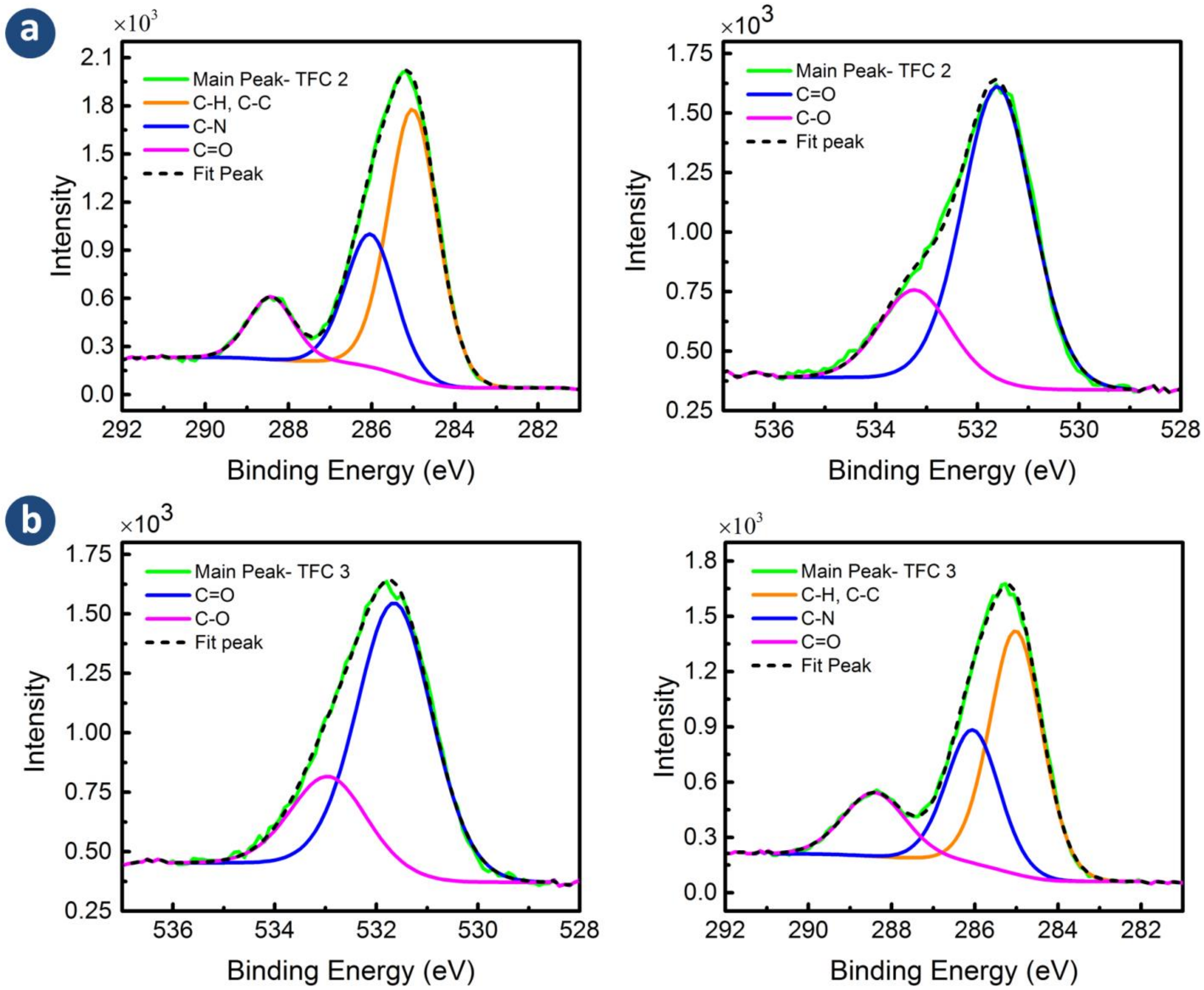

Figure S3: (a) convoluted high resolution C (1s) and O (1s) spectra of TFC 2, (b) convoluted high resolution C (1s) and O (1s) of TFC 3 membrane

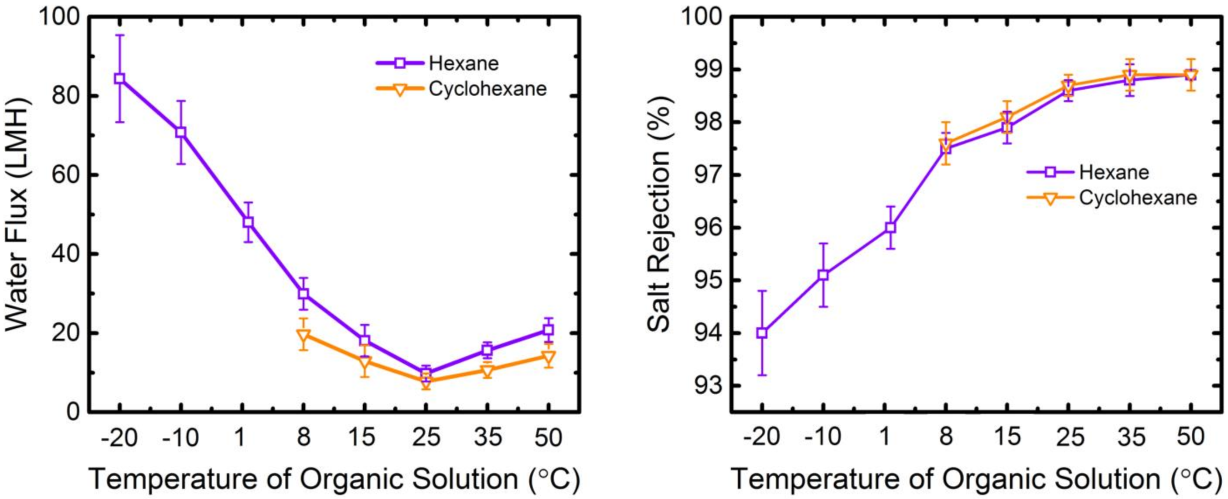

Figure S4: Water flux and salt rejection of the TFC membranes prepared at different temperature in hexane and cyclohexane solutions. Test conditions: feed solutions: pure water and 2000 ppm NaCl solution, pressure: 1.52 Mpa (220 psi), temperature: 25 °C, pH: 6.5-7

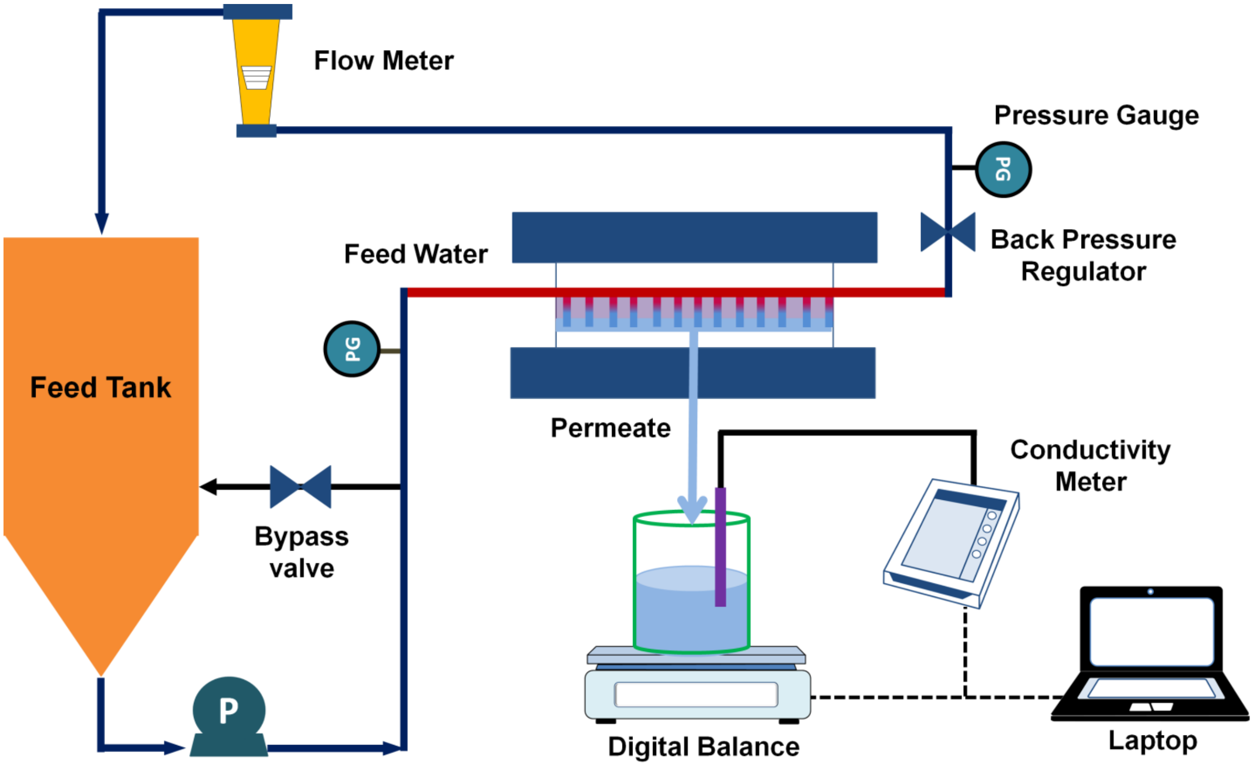

Figure S5: Schematic view of the cross-flow filtration setup used for characterizing the water permeability and salt rejection of the TFC membranes
